# Forecasting for the need of dentists and specialists in South Africa until 2030

**DOI:** 10.1371/journal.pone.0251238

**Published:** 2021-05-17

**Authors:** Ritika Tiwari, Ahmed Bhayat, Usuf Chikte

**Affiliations:** 1 Division of Health Systems and Public Health, Department of Global Health, Faculty of Health and Medical Sciences, Stellenbosch University, Cape Town, Western Cape, South Africa; 2 Department of Community Dentistry, University of Pretoria, Pretoria, Gauteng, South Africa; University of Western Australia, AUSTRALIA

## Abstract

To manage the increasing burden of dental diseases, a robust health system is essential. In order to ensure the oral health system operates at an optimal level going into the future, a forecast of the national shortfall of dentists and dental specialists in South Africa (SA) was undertaken. There is currently a shortage of dentists and specialists in SA and given the huge burden of dental diseases, there is a dire need to increase the number of these health care workers. The aim was to determine the projected shortfall of dentists and specialists in each of the nine provinces in SA. The projected shortfall was calculated based on the SA Disability-Adjusted Life Years (DALYs) for each province. The estimate for the evaluation of the Global Burden of Disease (GBD) for SA was obtained from the Institute of Health Metrics and Evaluation (IHME) Global Burden of Disease website. For each province, age standardized DALYs were calculated with mid-year population estimates obtained from Statistics SA 2018. In order to reduce the existing human resources for health (HRH) inequity among the provinces of SA, three scenarios were created focussing on attaining horizontal equity. The best-case scenario estimates a shortfall of 430, 1252 and 1885 dentists and specialists in 2018, 2024 and 2030 respectively. In an optimistic scenario, the national shortfall was calculated at 733, 1540 and 2158 dentists and specialists for the years 2018, 2024 and 2030 respectively. In an aspirational scenario, shortfalls of 853 (2018), 1655 (2024) and 2267 (2030) dentists and specialists were forecasted. Access to oral health services should be ensured through the optimum supply of trained dentists and specialists and the delivery of appropriate oral health services. Thus, the roadmap provided for upscaling the oral health services recognizes the influence of both demand and supply factors on the pursuit of equity.

## Introduction

Untreated dental caries was the most prevalent global condition which had affected 2.4 billion people [1]. In addition, untreated caries in deciduous teeth was the tenth most prevalent condition and had affected 621 million children worldwide [2]. To deal with this huge burden of disease, an effective and resourced health system is required. Health systems across the globe are dependent on the health workforce in improving health outcomes. The number of health professionals play a decisive role in the provision, monitoring and treatment of diseases. It is therefore essential to plan and forecast the required number of health professionals in order to meet the health-related goals nationally.

Currently, a mismatch exists between number of dental professionals and the populations served by them [3]. Over 93% of the World Health Organization (WHO) member states reported to have less than 1 dental personnel per 1000 population [4]. The majority of the world’s dentists are based in developed countries which has led to an inequitable distribution of human resources in dentistry; 69% of the world’s dentists serve 27% of the global population [5]. In Africa, the dentist-to-population ratio was approximately 1:150000 compared to 1:2000 for most developed countries; this further showcases the stark difference that exists globally [6]. Africa possesses only one percent of the global dental workforce and this could be a reason for the increasing burden of dental diseases on this continent [5, 7]. Additionally, immigration and emigration exacerbate this shortage as many dental professionals leave Africa and settle in more developed countries [5].

South Africa (SA) has an inadequate ratio of dentists per 1,000 population, and this together with a lack of access to oral health services, could be the driving forces in delayed diagnosis, untreated oral diseases and compromised health status [8, 9]. Within SA, many provinces have limited or no access to dental services at all [8]. Bhayat et al. reported that in 2016 there were 6 125 general dental practitioners and 481 specialists [10]. These specialists included 144 maxillofacial and oral surgeons, 142 orthodontists, 83 prosthodontists, 57 periodontists, 36 community dentists and 19 oral pathologists. The practitioner to population ratio for dental practitioners and specialists was 1:8 817 and 1:11 8947 respectively [10]. Although the study reported an overall increase of 27% in dental specialists from 380 in 2002 to 481 in 2015, there was still a dire shortage of dental professionals in the country [10].

In SA there are four dental schools which train dentists and dental specialists. The dental degree takes a minimum of five years to complete with an additional one year of compulsory community service. The specialist program takes a minimum of four years to complete except for oral pathology which takes a minimum of five years. These specialty training posts are government funded and only limited posts are available for their training.

In addition to the shortage of human resources, there is a maldistribution among the dental workforce. Approximately 20% of dentists are employed in the public sector which serves almost 80% of the total population of SA [11]. Also, stark imbalances have been reported in the quality of oral health care both inter and intra-provincially across SA [12].

The South African context, in terms of inequity, diversity, health and development challenges, provides a useful lens to examine HRH and development in UMICs (upper middle income countries) and LMICs (lower middle income countries). To plan for the ambitious reforms in delivery of oral health services in SA, valid and reliable information on the supply and need of qualified professionals are essential.

Disability-Adjusted Life Year (DALY) refers to one lost year of "healthy" life [13]. The sum of these DALYs across the population, or the burden of disease, can be thought of as a measurement of the gap between current health status and an ideal health situation where the entire population lives to an advanced age, free of disease and disability. DALYs for a disease or health condition are calculated as the sum of the Years of Life Lost (YLL) due to premature mortality in the population and the Years Lost due to Disability (YLD) for people living with the health condition or its consequences: DALY = YLL + YLD

DALYs suggest a health gap and their use has been suggested as a key measure of disease burden as it facilitates the comparison of different health problems across differing geographic areas [14]. Studies undertaken previously suggest a statistically negative relationship between the density of health workers and DALYs [15].

Thus, this study was undertaken to estimate the supply, need and projected shortfall for dentists and specialists at the national and provincial levels in SA using three different scenarios based on DALYs for oral disorders. These scenarios were created in order to reduce the existing HRH inequity within the provinces of SA. One model was based on the status-quo, the second model on achieving a provincial equivalence at 70 percentiles (to scale-up the last tertile i.e. 7^th^– 9^th^ rank) and the third model was based on achieving 40 percentiles (to scale-up the lower two tertiles i.e. 5^th^– 9^th^ ranks). Ethics approval was obtained from the Stellenbosch University Health Research Ethics Committee (HREC Reference No: X19/06/014).

## Materials and methods

### Forecasting future supply (2018–2030)

The number of dentists (including specialists) in each province was procured from the Health Professionals Council of South Africa (HPCSA) database (up to January 2018). Along with the already available workforce, HPCSA database includes new graduates, immigrants and professionals re-entering the profession. Based on provincial past trends from 2002 to 2017, the annual supply of dentists (including specialists) was forecasted. Using an Exponential Smoothing Technique (EST) (Single Exponential Smoothing, Alpha: 0.25), an advanced Microsoft Excel model, the extrapolated growth in the number of dentists and dental specialists from 2018 to 2030 was calculated. The EST is a time series forecasting method for univariate data that can be extended to support data with or without a systematic trend or seasonal component [16–18]. In addition, it is an intuitive forecasting method that weighs recent observations more heavily than earlier observations and uses transition probabilities in the predictive model using both their lower and upper 95% confidence limits [18].

Simultaneously, transition probabilities were explored in the predictive model using both their lower and upper 95% confidence limits.

### Forecasting future need (2018–2030)

For estimating and forecasting the need for services of dentists and dental specialists in a upper middle-income country like SA [19], the health need-based approach was adopted [20]. This analysis was carried out for each of the nine provinces based on DALYs as it represents ‘lost year of "healthy" life’ which plays a crucial role in determining the overall growth in a resource-deficit country like SA. Estimates for SA were obtained from the IHME GHDx website [21]. For each province, age standardized DALYs for oral disorders (caries of deciduous and permanent teeth, periodontal diseases, edentulism, tooth loss and other oral disorders) were calculated with mid-year population estimates obtained from Statistics SA 2018 [22]. The DALY load was calculated by dividing the provincial DALY score by the number of dentists and specialists registered in that province.

Setting equity-based targets: HRH norms for dentists per population are available [23]. However, since their applicability is not standardized as per lower or middle income countries, benchmarks were determined on the basis of DALYs per dentists (including specialists) for the different provinces in SA.

Estimating need for dentists: Global DALYs are projected to decrease by about 10% in absolute numbers from 2004 to 2030 [24]. Thus, it was assumed that the DALY load will decrease by a maximum of 10% for an upper middle income country (UMIC) like South Africa. The values from 2019 to 2029 were completed using an imputation method. The need for dentists (including specialists) were thus estimated using the DALY load per oral health specialists as estimated for benchmarking.

#### Forecasting dentists and dental specialists exiting the workforce

The number of dentists (including specialists) that were likely to exit the workforce were calculated based on retirement and migration.

*Retirement*. Assuming the retirement age to be 65 years, the outflow from the year 2018 to 2030 was calculated [25]. The age details were obtained from the HPCSA database.

*Migration*. There was no data available for the number of dentists (including specialists) migrating from SA. Hence, it was estimated, based on the net migration rate for SA, 0.9 people/1000 population would leave SA [26].

#### Gap estimation

Based on the HPCSA database [27], and on the knowledge and experiences of two authors in the study, the number of dentists (including specialists) were assumed to be 95% of the total number of registrations (which are currently active and working) for the baseline year 2018 [8]. For subsequent years, we estimated net workforce as:

$$\text{Net}\;\text{workforce}=\text{Number}\;\text{of}\;\text{dentists}\left(\text{including}\;\text{specialists}\right)\text{in}\;\text{health}\;\text{workforce}\left(\text{active}\;\text{and}\;\text{working}\right)+\text{supply}\;\text{of}\;\text{dentists}\left(\text{including}\;\text{specialists}\right)–\text{exiting}\;\text{dentists}\left(\text{including}\;\text{specialists}\right)\text{from}\;\text{workforce}$$

For consecutive years, the net workforce from the previous year was assumed to be number of dentists (including specialists) currently active and working in the workforce.

**Fig 1:**
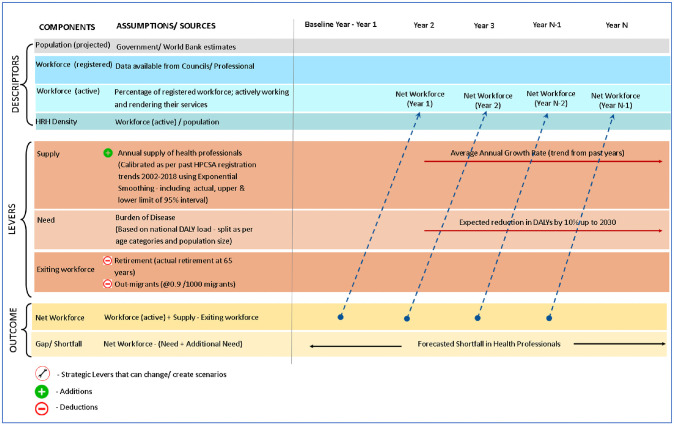
Workforce projection model.

This methodology has been adopted from similar HRH forecasting studies and is represented in Fig 1 [28, 29]. This figure explains the mechanism of the workforce projection model used for forecasting dentists. The model mentions the calculations undertaken for each province and includes the supply exit factors—retirement and emigration. The net workforce (active workforce + supply–existing workforce) for a particular year served as active workforce for the subsequent year.

*National shortfall*. To obtain a national projected dentist (including specialists) shortage, based on the province-wide forecasts, three scenarios were developed. The objective was to provide a roadmap for policy makers to try and reduce disparities related to oral health services within provinces in SA. The three scenarios and the assumptions for their equity targets have been explained in Table 1. DALYs for SA were projected to decrease by 10% up to 2030.(24) As a measure, the load of DALYs (for oral disorders) per dentist was calculated as DALY load. This DALY per dentist load was used for estimating the need of dentists and specialists.

**Table 1:** Shortfall of dentists (including specialists) as per three equity target based scenarios.

Scenarios	DALY load	Equity Targets
Scenario 1—Best Guess	Lower by 10% up to 2030	Status quo
Scenario 2—Optimistic (equivalent to province at 70 percentiles)		DALY/dentist load improved in three lowest-off provinces to level of the 6th province, ratios in top six provinces are maintained at 2019 levels. This target represents the scaling-up the last tertile of provinces.
Scenario 3 –Aspirational (equivalent to province at 40 percentiles)		DALY/dentist load improved in five lowest-off provinces to level of the 4th province, ratios in top four provinces are maintained at 2019 levels. This reflects a more ambitious target of eliminating inequality of the lower two tertiles of provinces.

Thus, the focus in this analysis is on horizontal equity which refers to providing equitable health care to all individuals [30].

### Projected change in national average of DALY load per dentists (including specialists) over years

**Fig 2:**
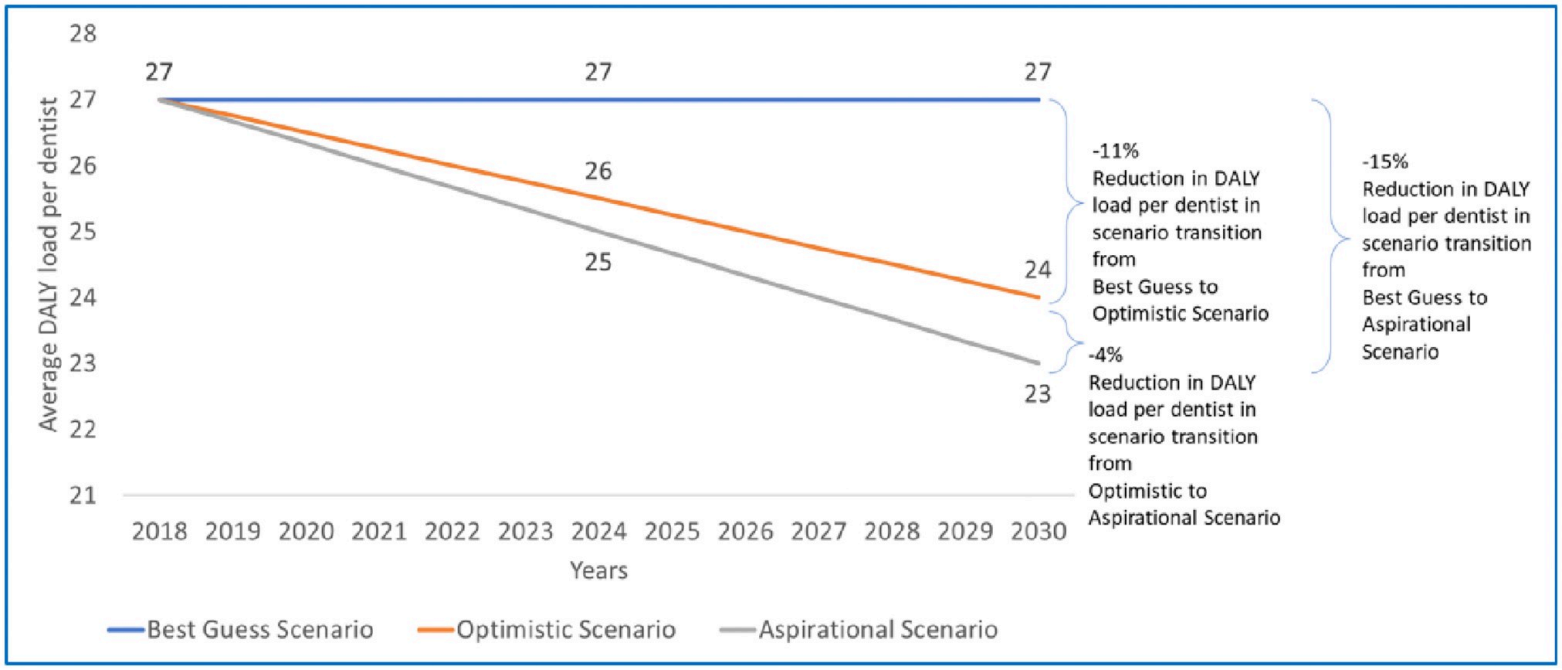
Change in national average of DALY load per dentists from 2018 to 2030.

Each equity-based scenario impacts the overall national DALY load per dentists and is expected to change if there is a transition within scenarios. Fig 2 explains the reduction in national average of DALY load per dentists for the three equity-based scenarios. In the case where the DALY load/dentist for the lowest three provinces is improved and made equivalent to the province at 70 percentiles, there will be an 11% average reduction in the DALY load/dentist. In the case where the lowest five provinces will have improved DALY/dentist load equivalent to the province at 40 percentiles then there will be a 15% average reduction in the DALY load/dentist. The aspirational scenario reduced the average DALY load by 4% in relation to the optimistic scenario.

## Results

Currently, within the South African context, along with dentists there are six categories of specialists (community dentists, maxillofacial and oral surgeons, oral pathologist, orthodontics, prosthodontics and periodontics) that offer oral health services. Almost half (40%) all dentists and specialists resided in Gauteng, 24% resided in Western Cape and 13% in KwaZulu Natal; less than 2% resided in the Northern Cape (Table 2).

**Table 2:** Geographical distribution of dentists and dental specialists in SA (January 2018).

Province	Dentists	Dental specialists						Total (%)
		Community Dentists	Maxillofacial and Oral Surgeons	Oral Pathologist	Orthodontics	Prosthodontics	Oral Medicine Periodontics	
Gauteng	2459	20	76	12	79	56	37	2739 (40.1%)
KwaZulu-Natal	859	3	18	-	13	4	4	901 (13.2%)
Mpumalanga	320	-	4	-	2	-	-	326 (4.8%)
Western Cape	1530	12	37	5	32	22	16	1654 (24.2%)
Limpopo	283	1	1	1	3	1	-	290 (4.2%)
Eastern Cape	350	-	8	-	7	2	2	369 (5.4%)
North West	215	1	1	-	3	-	-	220 (3.2%)
Free State	211	-	3	-	7	1	-	222 (3.2%)
Northern Cape	113	-	-	-	-	-	-	113 (1.7%)
**TOTAL**	6340	37	148	18	146	86	59	6834 (100%)

### Estimating and forecasting supply and need for dentists (including specialists) up to 2030

The DALYs for SA were obtained from the IHME GHDx website and the total load under the category “oral disorders for all ages” for the year 2017 was 122 310 [21]. The DALY load/dentist was calculated for each province based on their population and the registered number of dentists and specialists [22, 31]. Based on this, ranking was awarded to each province, i.e. the lower the DALY load/dentist (including specialists), the higher it’s ranking (Table 3).

**Table 3:** Province wide—DALYs, number of dentists (including specialists) and DALY load per dentists in SA.

Province	Population	DALYs	Number of dentists*	DALY load per dentist	Percentile	Rank
Western Cape	6621103	14029	1654	8	11%	1
Gauteng	14717040	31183	2739	11	22%	2
Northern Cape	1225555	2597	113	23	33%	3
KwaZulu-Natal	11384722	24122	901	27	44%	4
Free State	2954348	6260	222	28	56%	5
Mpumalanga	4523874	9585	326	29	67%	6
Eastern Cape	6522734	13820	369	37	78%	7
North West	3978955	8431	220	38	89%	8
Limpopo	5797275	12283	290	42	100%	9
**South Africa**	57725606	122310	6834	18	--	--

*Including specialists, excluding foreign category.

Thus, as per DALY load/dentists, the Western Cape, Gauteng and Northern Cape were the best performing provinces.

The Eastern Cape, Limpopo and North West were in the lowest provincial rankings, whereas the DALY/dentists load in Free State and Mpumalanga were in the moderate range as compared to the rest of the provinces.

#### Business as usual province-wide forecasts

**Fig 3:**
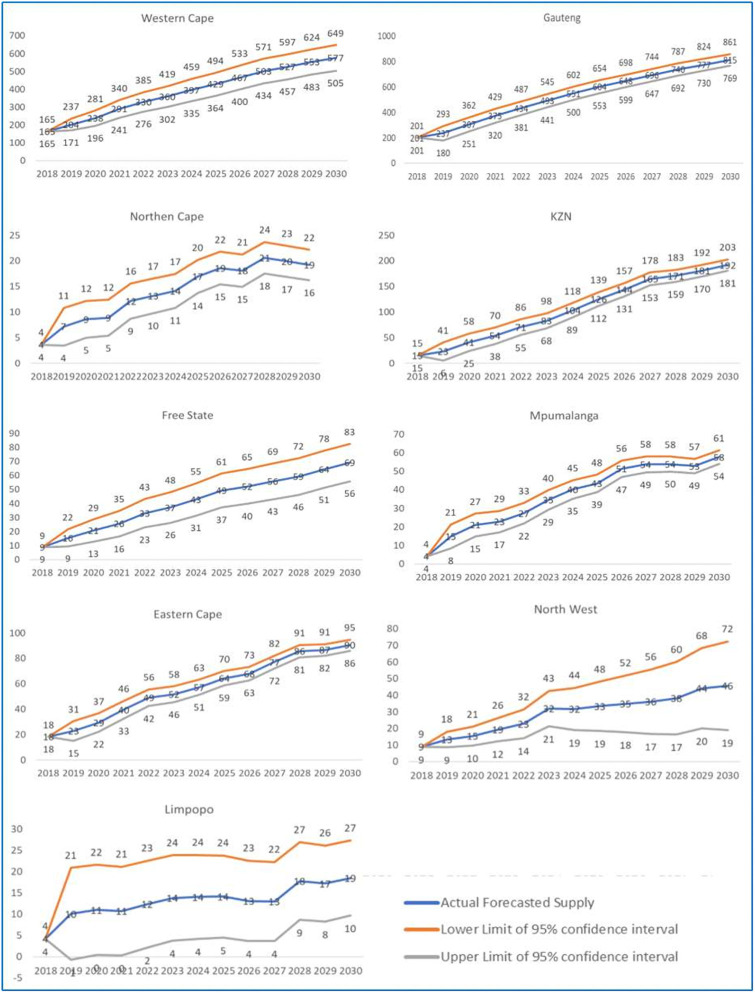
Forecasted province-wide shortage of dentists for SA (up to 2030).

Using the above methodology (Fig 1), the following province wide forecasts were generated for dentists (including specialists) up to 2030 in SA (Fig 3).

If the number of dentists and specialists continue to grow along the current trajectories since 2002 (business as usual), then the geospatial shortfall would be spread as per the past trends (based on geospatial inequity). For example, Western Cape might have a shortfall of 577 dentists (including specialists) as compared to Eastern Cape which may have a shortfall of just 90 dentists (including specialists) in 2030. Thus, despite the same population size their health needs are being decided on the basis of the past HRH trends, health facilities and infrastructure available in these provinces. It was imperative, therefore, to undertake a province-wide HRH forecasting exercise to showcase the existing geospatial inequity within the distribution and requirements of human resources among provinces within SA.

#### Scenario-based national forecasts

**Fig 4:**
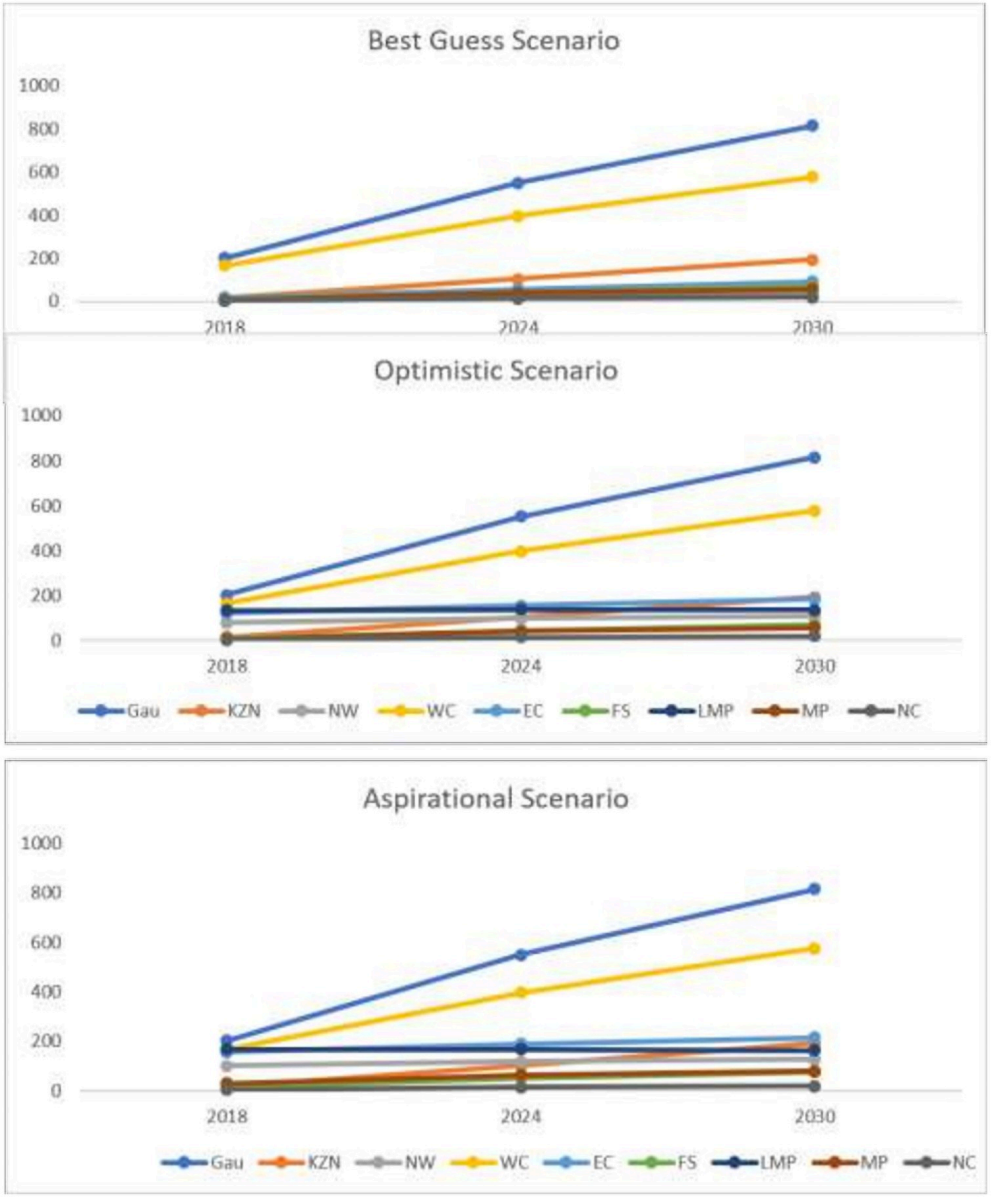
National shortfall for dentists and dental specialists based on three scenarios (up to 2030). Gauteng (GAU); KwaZulu-Natal (KZN); North West (NW); Western Cape (WC); Eastern Cape (EC); Free State (FS); Limpopo (LMP); Mpumalanga (MP); Northern Cape (NC).

**Fig 5:**
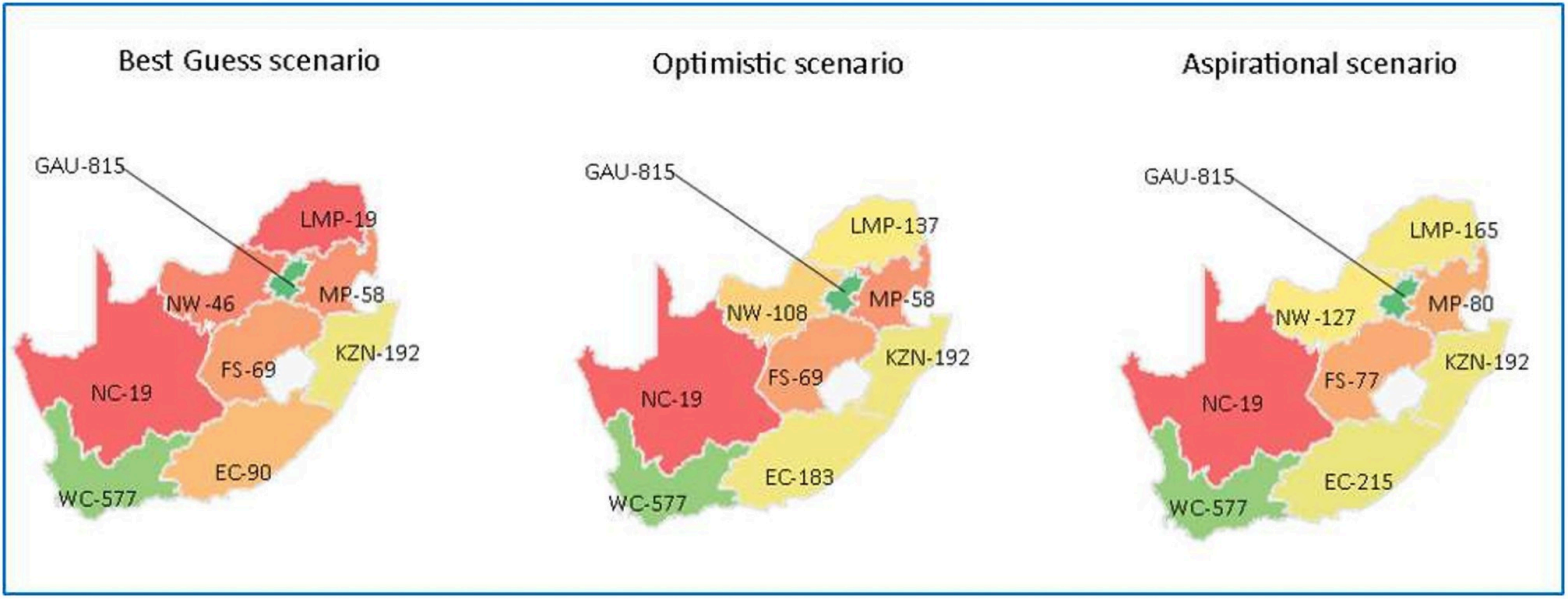
National shortfall for dentists and dental specialists per province based on three scenarios. Gauteng (GAU); KwaZulu-Natal (KZN); North West (NW); Western Cape (WC); Eastern Cape (EC); Free State (FS); Limpopo (LMP); Mpumalanga (MP); Northern Cape (NC).

Three scenarios based on reducing inequity in the number of dentists (including specialists) was calculated. The actual supply forecasts were considered and the upper and lower limit of 95% confidence interval not considered. The national shortfall of dentists (including specialists) in the three scenarios is provided in Table 4, Figs 4 and 5.

- Scenario 1—Best Guess: The shortfall for dentists (including specialists) was compiled province-wide to present a national figure of 430 (2018), 1252 (2024) and 1885 (2030).
- Scenario 2 –Optimistic: The shortfall in the lowest three provinces: Eastern Cape, North West and Limpopo; was estimated at the DALY/dentist rate of 70 percentiles (i.e. Mpumalanga 29 –Rank 6). Thus, nationally a shortfall of 733, 1540 and 2158 dentists (including specialists) were forecasted for 2018, 2024 and 2030 respectively.
- Scenario 3 –Aspirational: The shortfall in the lowest three provinces (Eastern Cape, North West and Limpopo) and two moderate provinces (Free State and Mpumalanga) were estimated at the DALY/dentist rate of 40 percentiles (i.e. KwaZulu-Natal 27 –Rank 4). Thus, at national level a shortfall of 853, 1655 and 2267 dentists (including specialists) were forecasted for the years 2018, 2024 and 2030 respectively.

**Table 4:** National shortfall for dentists and dental specialists based on three scenarios (up to 2030).

Provinces	Scenario 1—Best Guess			Scenario 2—Optimistic			Scenario 3—Aspirational		
Year	2018	2024	2030	2018	2024	2030	2018	2024	2030
Gauteng	201	551	815	201	551	815	201	551	815
KwaZulu Natal	15	104	192	15	104	192	15	104	192
North West	9	32	46	78	97	108	99	118	127
Western Cape	165	397	577	165	397	577	165	397	577
Eastern Cape	18	57	90	121	155	183	157	189	215
Free State	9	43	69	9	43	69	17	51	77
Limpopo	4	14	19	135	139	137	167	168	165
Mpumalanga	4	40	58	4	40	58	29	63	80
Northern Cape	4	14	19	4	14	19	4	14	19
**Total**	**430**	**1252**	**1885**	**733**	**1540**	**2158**	**853**	**1655**	**2267**

These graphs represent the requirement for dentists and dental specialists at a national level for 2020, 2024 and 2030. For provinces with a higher population load such as Western Cape, Gauteng and KwaZulu Natal–the need will also be higher. However, the idea is not to reduce their needs but provide equitable access to care and oral health services to least performing provinces and increasing their needs to the levels of better performing provinces to achieve horizontal equity.

These maps represent the requirement for dentists and dental specialists at national level for 2030. As we move from left to right, the horizontal equity increases and thus the need for dentists and specialists increases within the provinces. The green colour denotes more requirement vs red colour which denotes a lesser requirement. The focus is to bring the least performing provinces into the green zone i.e. to increase their requirement for dentists and dental specialists for better availability of workforce for achieving universal health coverage of oral health services in 2030.

## Discussion

Globally, oral health disparities continue to widen, more in the disadvantaged and vulnerable groups where the burden of oral diseases is the highest [32–34]. In SA this is exacerbated by a shortage of health facilities, inadequate workforce and the unequal distribution of these services in the country [35]. Furthermore, due to the quadruple burden of disease including Human Immunodeficiency Virus (HIV), Tuberculosis (TB), maternal and child mortality and non-communicable diseases (NCDs), the funding for oral health is limited [36]. Almost 90% of the South African population was dependent on the public oral health services, whereas in 2009 only 25% of all South African dentists were employed within the public sector [37]. As a result, there is a need for an alternative oral health care system. In contrast to the existing curative-driven and individually focused system, the new system should be population-based focusing on prevention of oral disease and oral health promotion. This may require the expertise of oral health specialists, academics and public health experts [37].

Oral health has not been addressed in SA effectively. Clinical services related to the treatment of oral health conditions are expensive in terms of human resources and dental equipment for the population [38] and hence the focus should be onprevention rather than on curative care [39].

This preventive approach has shown to be effective in developed countries which have reported significant savings in dental health expenditure and reductions in the prevalence of oral diseases [37] In SA, the role of the dental public health specialist is largely restricted to academic institutions and research. If these specialists work in collaboration with relevant stakeholders to develop policies to plan, implement, monitor and evaluate oral disease prevention programs, SA could have a better prospect of reducing its oral health disease burden [37].

Since many aspects of oral health education and employment in SA are unknown, a series of assumptions was necessary. For example, reasons for the workforce attrition, the adjustment for the different lengths of courses and the possibility of individuals working in both public and private sectors and any change in the technology (increasing efficiency) of current dental services were not taken into account for the projections.

This study is based on DALYs for oral disorders which included caries of deciduous and permanent teeth, periodontal diseases, edentulism and other oral disorders. However, it did not incorporate the analysis for other oral health professionals such as, oral hygienists and dental therapists which contribute to the diagnosing, treatment and management of oral diseases in SA. There is a need for further analysis incorporating factors such as health facility distribution, skill mix and team size for oral health professionals.

Notably, the Immigration Regulation of 2014 repealed the ’Exceptional Skills’ Visa, with the current ’Critical/ Scarce Skill’s Visa omitting dentistry from this category [40]. Thus, foreign born dentists, including those with South African qualifications, face challenges while immigrating and have to explore other means of securing work permits in SA.

It is clear that disparity exists in distribution of dentists and dental specialists as well as the services being offered to populations within the provinces. For instance, the population size of Western and Eastern Cape is nearly equivalent but on the basis of DALY load/dentist; the Western Cape is the best ranked province whereas Eastern Cape is ranked the lowest. This impacts on the availability of health infrastructure and related oral health services.

Thus, to improve the status and number of dentists and specialists in SA a set of policy interventions would be required. The first step would be to identify that there is a problem and be incorporated into the National Department of Health’s (NDoH) future strategy for HRH in SA. In addition, there should be a need to create health infrastructure and positions for oral health specialists at national, provincial and district level. Lastly, with regards to career pathways for dentists and specialists; these projections will only likely to be realized through concerted advocacy and action in several areas by policy-makers and NDoH in SA.

SA is characterized by high wealth inequality and low intergenerational mobility which arise from high income inequality and inequality of opportunity for children. With a consumption expenditure Gini coefficient of 0.63 in 2015, SA is one of the most unequal countries in the world with incomes being highly polarized [41]. Since SA is adopting the National Health Insurance (NHI), availability and access to oral health services as a part of the health care package must be made universal. The barriers to utilization, such as the availability of resources, long waiting times and user fees must be made equitable to populations belonging to all economic classes and provinces. The equity of access principle should apply on both sides, the supply of human resources into the system and the provision of preventive and curative care. Thus, the roadmap provided for upscaling the oral health services recognizes the influence of both demand and supply factors on the pursuit of equity.

## Conclusion

There is an imbalance in terms of the inter-provincial, inter-sectoral and urban versus rural distribution of dental practitioners. There is an additional inequity in access between the public and private sectors. Overall, ‘a fit for purpose’ oral health workforce with equitable and improved health outcomes can be enabled. No matter which case scenario is achieved, there will be a shortage of dentists and specialists in SA which will range from 1252 to 1655 in 2024 and increase from 1885 to 2267 in the year 2030. If the current scenario is not rectified, then the majority of the population will continue to endure inequities in terms of accessing efficient and effective oral health services.

## Recommendations

The number of dentists and specialists should be increased to deal with the increasing needs of the communities.

Compulsory community service should be limited to rural areas to address the maldistribution of human resources and disease burdens.

Essential packages of oral health interventions need to be provided in school and community settings to improve people’s control over their oral health and prevent oral diseases.

Health financing schemes and inclusion of dental insurance programs within the NHI package of services is essential to improve oral health especially in rural communities. There should be a provision for covering the costs of oral health care, with focus on integrated disease prevention, health promotion and minimally invasive treatment.

The NDoH and other policy makers can utilize this study as a roadmap for providing equitable access to care and oral health services. The NDoH needs to address both the shortfall in the number of dentists and specialists and the inequitable distribution in order to reduce the existing burden of disease within the provinces of SA.
